# Analysis of longitudinal sections of retinal vessels using Doppler OCT

**DOI:** 10.1364/BOE.385938

**Published:** 2020-03-04

**Authors:** Sylvia Desissaire, Florian Schwarzhans, Matthias Salas, Andreas Wartak, Georg Fischer, Clemens Vass, Michael Pircher, Christoph K. Hitzenberger

**Affiliations:** 1 Center for Medical Physics and Biomedical Engineering, Medical University of Vienna, Vienna, 1090, Austria; 2 Institute of Medical Information Management, Medical University of Vienna, Vienna, 1090, Austria; 3 Wellman Center for Photomedicine, Harvard Medical School and Massachusetts General Hospital, Boston, Massachusetts 02114, USA; 4 Department of Ophthalmology and Optometry, Medical University of Vienna, Vienna, 1090, Austria

## Abstract

We present a new method for imaging retinal vessels that provides both structural and hemodynamic information. Our technique is based on a single beam OCT system with an integrated retinal tracker that enables recording of arbitrary scan patterns. We record longitudinal sections along the traces of retinal vessels. The tracker function enables the acquisition of multiple longitudinal sections along the same trace to provide high-quality averaged OCT scans as well as temporal changes of flow dynamics. The vessel walls are clearly identified as narrow, bright lines from which the vessel diameter can be retrieved as a function of position along the vessel. Furthermore, the Doppler angle can be obtained at each position along the vessel trace, enabling measurement of absolute blood flow by Doppler OCT analysis. The method is demonstrated in flow phantoms and *in-vivo* on retinal vessel bifurcations in healthy volunteers. In 7 of 9 imaged bifurcations, measured in- and outflow deviate by less than 11%, demonstrating the consistency of the method.

## 1. Introduction

Alteration of retinal blood flow (RBF) is known to be associated with various ocular diseases such as glaucoma or diabetic retinopathy (DR) [1–4]. Further investigations on the interaction between RBF and retinal diseases and their progression may help to improve early diagnostics and treatments. Different methods have been developed to visualize ocular vasculature and to quantify blood flow in the living human eye. Label free methods are particularly suitable as they are non-invasive and free from possible adversary effects, thereby enabling convenient monitoring of the changes during the course of disease development.

Optical coherence tomography (OCT) angiography (OCTA), a functional extension of OCT, has found widespread use for label free imaging of retinal vasculature in recent years [5–7]. OCTA exploits the variations in the intensity and/or phase of the signals caused by motion of blood cells, which allows to differentiate vasculature from static tissue. While this method provides valuable information on the structure of retinal vasculature, quantitative information on blood flow cannot be directly retrieved. A related method, Doppler OCT (DOCT) [8] can provide quantitative information on the velocity of the moving blood constituent. DOCT typically records cross sectional images of a blood vessel. By recording two scans at the same position with a time delay in between, the axial velocity component of a moving blood cell can be retrieved from the phase difference of the signals [8]. However, to obtain the absolute velocity, the Doppler angle (angle between velocity vector and probing beam) has to be known.

Different approaches to solve the Doppler angle problem have been reported to date. One solution is to obtain additional information on the vessel orientation. This can be achieved by recording cross sections in two planes separated by a small distance along the vessel length [9], whereby the angle of the vessel with respect to the imaging beam can be derived from the position of its cross-section in the two B-scans. This method was extended to record the total blood flow through the vessels emerging from the optic nerve head (ONH) by recording two circumpapillary scans around the ONH with different radii of the scan trace [10,11]. One shortcoming of this method is the assumption of a straight vessel segment between the two sections. Another option that avoids this assumption is to retrieve the vessel geometry from full 3D data sets [12,13]. However, this method is sensitive to errors that can be induced by sample motion during the recording of the 3D data set. Since the Doppler angle of retinal vessels is usually close to $90^{\circ }$, even slight distortions of the Doppler angle will have a large influence on the measured flow. Another approach derived the blood flow without explicit knowledge of the full velocity vector. This method can only be applied to vessels with small Doppler angles as is the case for those located close to the nerve head [14]. By integration of the measured axial flow components over the elliptical cross section of the vessel retrieved from a volume scan, the flow can be determined. However, this technique requires high imaging speeds of hundreds of kHz A-scan rates and residual motion within the data recording will influence the measured elliptical cross section and thus the measured flow.

To overcome these problems, multi-beam OCT systems were developed. First versions used two imaging beams [15–17] illuminating the sample at two different angles, thus providing two components of the velocity vector. Similar systems successfully demonstrated the measurement of absolute blood flow velocity in human retinal vessels [18,19], however, usually require the knowledge of the azimuthal vessel orientation in the image plane which can be taken from an en-face fundus image, or assuming a radial vessel orientation near the ONH. A full reconstruction of the flow velocity vector without the need for any prior knowledge on the vessel orientation was finally provided by three-beam DOCT systems that illuminate the retina at three different incident angles simultaneously [20]. Different variants of this method have been reported for both spectrometer based systems [20,21], as well as for swept source based systems [22,23]. While these methods were successfully demonstrated to provide absolute velocity and total blood flow measurements in the human retina with a precision of down to ∼ 6 %, their common drawback is a considerably larger complexity of the system and alignment procedures.

In this work, we report on a new approach to measure absolute blood velocity and flow in retinal vessels. It is based on an OCT system combined with a line scan laser scanning ophthalmoscope (LSLO) that enables high precision retinal tracking [24], whereby the tracking follows a concept that had been introduced earlier [25,26]. Consequently, only a single OCT sampling beam is needed. The system allows recording of arbitrary scan traces. Contrary to conventional DOCT, we don’t record vessel cross sections; instead, we record longitudinal sections of a vessel along its trace. The retinal tracker enables repeated measurements along exactly the same trace, providing high-quality structural images as well as images of the axial velocity component. From these images, the Doppler angle and the vessel diameter can be calculated at every position along the vessel trace, and quantitative velocity and flow data can be extracted. We demonstrate the method in a flow phantom and provide measurements of blood flow in artery and vein bifurcations of healthy human subjects.

## 2. Methods

### 2.1. Experimental set-up

The setup used for imaging is an SD-OCT system with an integrated retinal tracker, as described in previous literature [24]. The OCT subsystem is based on a Michelson interferometer with polarization maintaining (PM) fibers (this instrument can also be used for polarization sensitive imaging, a function not used in this work). A superluminescent diode (SLD) with a center wavelength of 860 nm and a bandwidth of 60 nm is used for imaging at an A-scan rate of 70kHz. The resolution achieved is 4.2 μm axially (in tissue) and 20 μm laterally (the axial resolution has been determined with a mirror as the sample and measuring the full width at half maximum of the axial point spread function while the lateral resolution has been derived from the imaging beam diameter). The sensitivity of the system is 98 dB. Dimensions of the usual raster volume acquired are 8 mm (x direction) x 6 mm (y direction). A LSLO is used for retinal tracking in real time. This subsystem operates with a laser light source at a center wavelength of 786 nm and illuminates the retina with a line shaped beam of 8 mm x 20 μm. The power to the eye is 0.7 mW from the LSLO and 0.5 mW from the OCT beam, which satisfy the laser safety regulations. In the previous version of the instrument [24] the tracking procedure was based on a subimage (of usually 64x64 pixels) of the LSLO template that was manually selected to contain high-contrast structural features like vessel bifurcations or crossings. Retinal motions were identified in real time by cross-correlation with the real time LSLO images, the output from this cross-correlation was used to accordingly offset the x and y OCT scanners positions during acquisition to compensate for the motion artifacts in real time. In order to improve the tracking performance, this method was modified to consider the full LSLO image (800x600 pixels) as the template for cross-correlation. Thus, the acquisition procedure is simplified as manual selection is no further needed. The method shows to be more reliable especially in locations without high-contrast structural features, such as the temporal region, as compared to the previous sub-image based tracking method, since with the full frame more overall information is available for determining the displacement introduced by eye motion. A new GPU-based algorithm was developed to achieve the same update rate of the scanners offset as previously, despite the larger amount of calculation.

### 2.2. Recording of longitudinal vessel sections

**Fig. 1.**
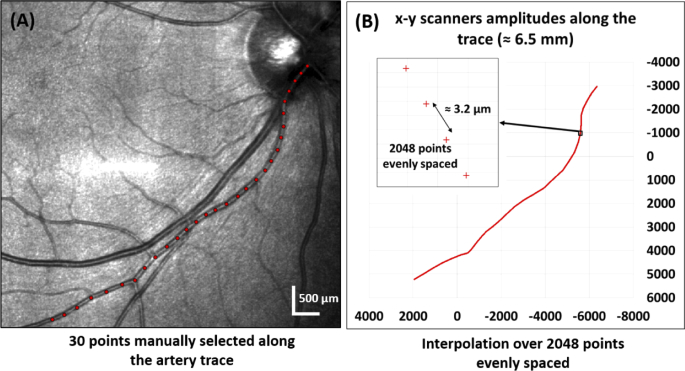
Example of selecting the trace for a longitudinal vessel section from the LSLO template of the right eye of a healthy volunteer. (A) LSLO reference frame with the manually marked points along the trace in red. (B) Graph of the trace coordinates to control the OCT x and y scanners after interpolation of the marked points and conversion to scanner amplitudes.

In this work, longitudinal sections of individual vessels are recorded, with the scanning OCT beam following exactly the vessel trace. To select the vessel trace, an averaged LSLO image (reference frame that is used for retinal tracking) is used, and the following steps are performed:

*Step 1.* Several points (usually between 15 and 30) along the vessel are manually marked on the LSLO reference frame as shown in Fig. 1(A). These should be located as close as possible to the center of the vessel and do not need to be evenly spaced. The point spacing is usually denser at vessel curvatures as compared to linear parts.

*Step 2.* In between the marked positions, linear interpolation is carried out to distribute the selected number of A-scans (usually 1024 or 2048), as shown in Fig. 1(B). The 1024 or 2048 positions obtained are evenly spaced along the trace.

*Step 3.* The coordinates of the points are converted to input amplitudes for the x and y OCT scanners and correspond to each A-scan location. The scanning beam can then be moved along the trace.

*Step 4.* Before measurement, the tracking is turned on to correct for subject motion in real time. Repetitive B-scans (typically 100-200), each along the same vessel trace, can then be acquired over several cardiac cycles.

### 2.3. Measurement protocol

#### 2.3.1. In-vitro measurements

In a first step, *in-vitro* measurements at different flow rates were performed to demonstrate the accuracy of the quantitative measurements. A flexible plastic capillary of internal diameter 280 μm (330 μm when filled with liquid due to the pressure on the capillary walls) was taken as the phantom. The capillary, fixed on a mount, was curved both in axial (to generate different Doppler angles) and transverse (to mimic vessel curvature for trace selection) directions. An additional lens of focal length 30 mm was used for imaging (as the system is designed for retinal imaging). The size of the recorded images was calibrated using a USAF 1951 resolution test target. The capillary was perfused with a scattering medium consisting of milk diluted in water (ratio 1:3). Different flow rates were set using a syringe pump (Legato 200, possible flow rate ranging from 3.06 pl/min to 215.8 ml/min with an accuracy of ±0.35%). The mean diameter of major retinal vessels is typically ∼ 130 μm for arteries and ∼ 150 μm for veins in healthy subjects [27]. In order to reach the same velocity range as for *in-vivo* imaging [28,29], the capillary was perfused at increasing infusion rates ranging from 40 μL/min to 140 μL/min with an increment of 20 μL/min. 100 B-scans each consisting of 1024 A-scans were recorded during each acquisition, i.e. at each rate value.

#### 2.3.2. In-vivo measurements

Seven healthy volunteers aged 29 to 36 years were imaged for this study after informed consent was obtained. The study was approved by the university’s ethics committee and is in agreement with the tenets of the Declaration of Helsinki. For five subjects, both an artery and a vein bifurcation were selected and imaged to demonstrate the precision of the method *in-vivo*. In-flow into the bifurcation is expected to match the total out-flow from the bifurcation. Measurements were repeated 3 times along each of the two traces of each of the bifurcations. The volunteer was realigned in between the individual measurements and the LSLO template and vessel traces were re-selected. 200 B-scans of 1024 A-scans were repeated at the same location along the vessel. The vessels were imaged along a length of approximately 3 to 4 mm. Imaging along a longer (around 6 mm) artery and vein trace was carried out in two different subjects. For these measurements, 200 B-scans of 2048 A-scans each were recorded. Measurements were carried out in the direction of the blood flow (similar results are obtained when imaging in the opposite direction).

### 2.4. Flow evaluation

#### 2.4.1. General method

**Fig. 2.**
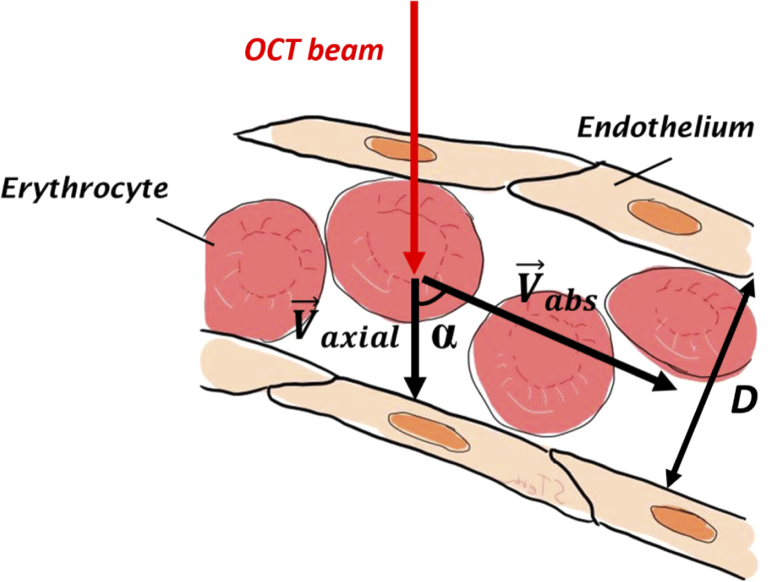
Schematic of a vessel with $V_{abs}$ the absolute velocity, $V_{axial}$ the axial velocity, α the Doppler angle and D the vessel diameter.

From the acquired longitudinal vessel sections, we can retrieve the absolute blood velocity, and consequently the absolute blood flow in the following way (cf. Fig. 2):

After standard Fourier domain OCT signal processing, intensity tomograms and Doppler (phase difference between consecutive A-scans) tomograms are calculated. Axial velocity along each A-scan of each B-scan is then calculated as:

(1) $$V_{axial}(z)=\frac{\Delta \varphi (z)*\lambda_{0}}{4\pi *n*T}$$

with Δφ the phase difference at depth position z between consecutive A-scans, $\lambda_{0}$ the center wavelength of the illumination light, n the refractive index of the medium and T the inverse A-scan rate. After segmentation of the vessel walls and center, its diameter D and the Doppler angle α can be retrieved at each A-scan position. The absolute velocity is then obtained by:

(2) $$\left|{\vec{V}}_{abs}(z)\right|=\left|\frac{{\vec{V}}_{axial}(z)}{\cos\alpha }\right|$$

The maximum velocity $V_{max}$ is obtained by a second order polynomial fit to the velocity profile $V_{abs}(z)$. Assuming a circular vessel cross section, the blood flow Q can then be obtained by:

(3) $$Q=\frac{V_{mean}*\pi *D^{2}}{4}$$

with:

(4) $$V_{mean}=\frac{V_{max}}{2}$$

#### 2.4.2. In-vivo flow measurements

*Step 1: Vessel segmentation based on averaged intensity images*

**Fig. 3.**
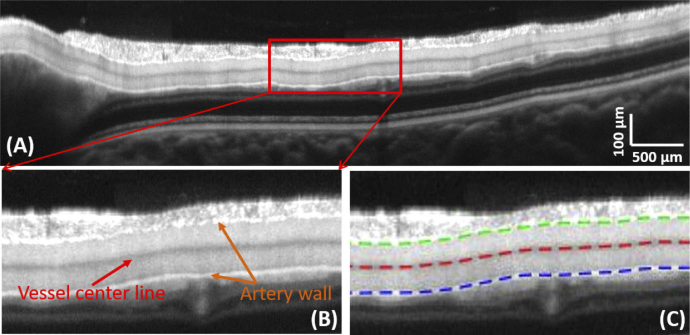
Vessel segmentation based on the averaged longitudinal section scan. (A) Averaged intensity image (over 100 B-scans) from a longitudinal vessel section along an artery of the right eye of a healthy volunteer. The vessel with its bright upper and lower vessel walls and its dark center line can easily be distinguished from the surrounding tissue. A zoomed image of the framed area in red is given in (B) and (C), without and with the segmentation lines, respectively.

Intensity tomograms are registered to each other and averaged to produce high quality sectional images along the vessel trace. The vessel shape is retrieved from an averaged image over 100 B-scans (cf Fig. 3). The upper and lower wall of the vessel as well as a dark center line can be distinguished and segmented (cf. Fig. 3(B) and (C)). Segmentation is performed semi-automatically. First, the vessel walls and the dark center line are manually segmented on the averaged intensity image. A graph-based method is then used to improve the precision of the segmentation of the vessel walls. The method is based on a previously published algorithm [30]. For each A-scan, the boundary of steepest change from dark to bright is detected. Starting from the manual segmentation of the dark center line, the algorithm searches for the upper and lower vessel walls on the averaged intensity image. Insufficient image quality and irregularities at the vessel walls along the trace, as for example the discontinuities at bifurcations, may however cause erroneous segmentation results. At these locations, the segmented points are removed (erratic locations are defined as a deviation from the manually segmented wall larger than ±5 pixels i.e. ±7 μm in depth). The resulting segmentation lines are finally smoothed to extract the wall positions at each A-scan. The dark center line is defined as the pixel location of lowest intensity value in between the vessel walls and the resulting trace is also smoothed.

*Step 2: Calculation of absolute velocity tomograms along the vessel trace*

The Doppler tomograms were registered (x-z-translation only) and the phase difference values were bulk motion corrected. The Doppler angle values at each A-scan position are calculated from the center line segmentation only. As the shape of the vessel may vary somewhat with position, the Doppler angle values may accordingly slightly vary. However, these variations were here considered negligible. Vessel sections that have Doppler angles ranging from $85^{\circ }$ to $90^{\circ }$ (i.e. when the inclination of the vessel wall is close to perpendicular to the imaging beam and the vessel appears in horizontal direction in the B-scan image) are excluded from quantitative evaluation to avoid excessive errors by a division with a value close to zero. To reduce the noise, the final Doppler tomograms are calculated as floating average over five consecutive Doppler tomograms (B-scan ±2).

*Step 3: Blood flow quantification*

The blood flow values calculated in this study are averaged over several B-scans, usually including 2 to 4 full cardiac cycles for the arteries (from systolic peak to systolic peak) and over the full sequence for the veins. Thereby, the total number of B-scans varied between the subjects depending on the heartbeat rate and on the fixation capability of the individual as image frames containing larger motion artifacts have been excluded from the analysis. For each velocity tomogram, a vessel section (window) containing at least 100 A-scans is selected. The flow is extracted from the data in this window. For each A-scan position in this window, flow is calculated as described in section 2.4.1. The final flow value is calculated as the average of the single flow values of these selected A-scans and averaged over the selected B-scans.

## 3. Results

### 3.1. Phantom measurements

**Fig. 4.**
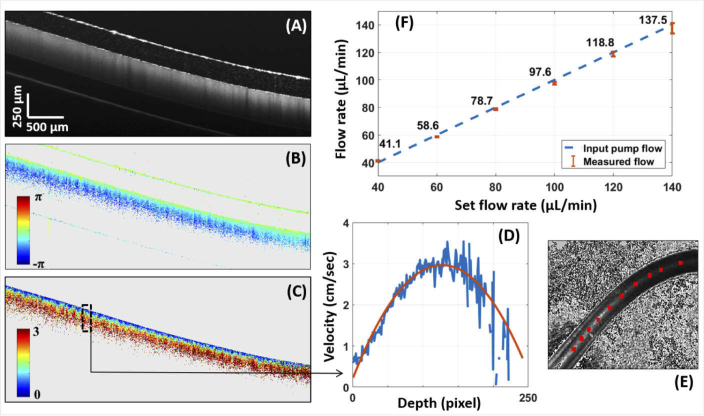
Flow measurements in a phantom. The plastic capillary was imaged and analysed by DOCT while perfused with different input flow rates. (A), (B) and (C) are respectively the averaged OCT intensity scan, the phase difference (between A-scans) scan and the absolute velocity scan at an input flow rate of 80 μL/min. An averaged velocity profile, at the location marked in (C), is given in (D). The graph (F) plots the measured flow rate compared to the input flow values defined by the pump. The standard deviation values are calculated between the averaged flow values of each B-scan. The LSLO image of the capillary is given in (E).

Figure 4(A) shows an averaged OCT intensity image of the longitudinal section of the plastic capillary. The corresponding LSLO image is given in Fig. 4(E). The averaged calculated diameter is 330 μm when perfused with liquid. The Doppler angle varies from $78^{\circ }$ to $84^{\circ }$, corresponding to the values observed in retinal vessels. Phase difference as well as absolute velocity tomograms at a flow rate of 80 μL/min are shown in Fig. 4(B)-(C). We observe changes in phase differences along the capillary according to the Doppler angle. These differences vanish after computing the absolute velocity tomogram. A flow profile can be plotted at each location, an example is given in Fig. 4(D) as an average over 50 consecutive A-scan positions. As expected, a parabolic velocity profile is observed. The larger noise in deeper areas of the flow phantom is caused by the reduced signal due to the strong attenuation in the scattering liquid. The orange line shows the result of a second order polynomial fit. The measured flow values are in the range of the input values of the pump as shown on the graph in Fig. 4(F) with an error of approximately 2-3 %.

### 3.2. *In-vivo* measurements: structural imaging

**Fig. 5.**
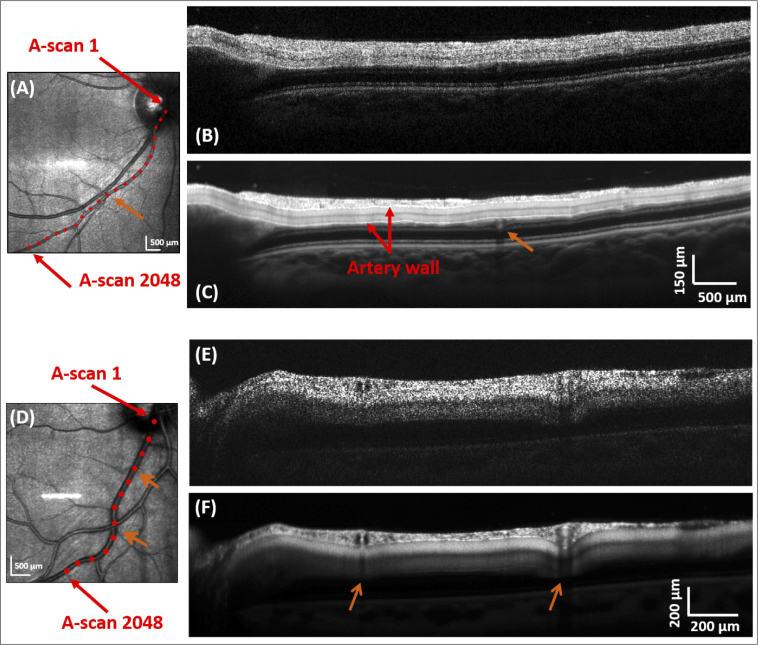
Imaging along the trace of a retinal artery (A)-(C) and vein (D)-(F) of two healthy volunteers as shown in the two LSLO templates (A) and (D). (B) and (E) are single OCT intensity scans and (C) and (F) are the averaged OCT intensity scans over the 100 best correlated B-scans. Vessel crossings are marked by the orange arrows.

Figure 5(A)-(C) shows a LSLO image (A) and intensity OCT tomograms (B),(C) recorded along a 6.5 mm long segment of a retinal artery starting at the ONH (same eye as in Fig. 1). The mean artery diameter is about 80 μm. 2048 A-scans per B-scan were recorded. Figure 5(B) is a single scan of the acquisition while Fig. 5(C) is an average over the 100 best correlated scans after registration. A video of the full sequence of the acquired intensity tomograms is given in the supplementary material (Visualization 1). Saccades occurring during image recording can be identified as "flashing frames" throughout the sequence. Speckle structures can still be seen in the RNFL of the averaged image indicating a good tracking performance. Below the RNFL, we observe the upper and lower wall of the vessel, recognizable as a bright line in the image. The artery wall can clearly be resolved both on the averaged and single scan. Inside the vessel, the intensity is homogeneous apart from a dark line in the center. Such a dark line was not observed in the phantom measurements but is repetitively seen *in-vivo*. This reduced intensity may be due to the orientation and deformation of red blood cells (RBCs) in shear flow. It corresponds well to previous observations reported in literature on the signal intensity from within a vessel [31–33]. Since RBCs have a biconcave shape, distribution of light scattering is dependent on illumination position of the cell (e.g. rim vs. face of the cells [34]). The location of a small vein crossing beneath the artery can also be identified, as indicated by an orange arrow on both the LSLO image (Fig. 5(A)) and the averaged intensity image (Fig. 5(C)).

Figure 5(D)-(F) shows results for the imaging of a vein of mean diameter of about 160 μm. Similarly to the artery, we observe the vessel wall and the dark center line. Two locations of crossing with other retinal vessels are clearly visualized, at 600 μm and 1.4 mm away from the ONH (marked by two orange arrows).

In order to demonstrate the repeatability of the method of longitudinal vessel imaging and blood flow quantification, both an artery and a vein bifurcation of five additional healthy volunteers were imaged and analysed. For each bifurcation, three acquisitions of each of the two traces were taken.

**Fig. 6.**
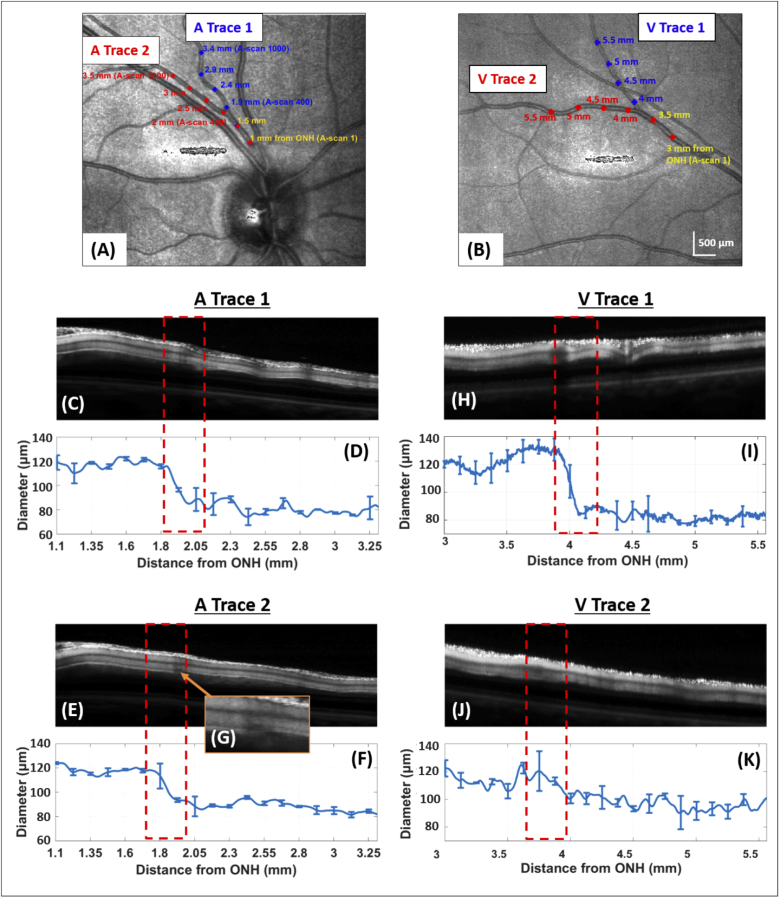
Imaging along both an artery and vein bifurcation of the right eye of a healthy volunteer. LSLO images with the plotted traces of two imaged bifurcations - (A) artery and (B) vein - are shown. In the center of the LSLO images is an imaging artefact. (C) and (E) as well as (H) and (J) are the averaged intensity tomograms of the two longitudinal sections of respectively the artery and vein bifurcation. (D) and (F) as well as (I) and (K) are the corresponding graphs of the vessel diameter along its length. Location of bifurcation is highlighted by the red dashed rectangle. (G) is a zoom-in on the artery bifurcation area, where irregularities of the lower vessel wall can be seen.

Figure 6 shows the example of an artery and a vein bifurcation of one subject. The LSLO images of Fig. 6(A)-(B) depict the analysed vessels ((A) artery, (B) vein) and their position on the ocular fundus, Fig. 6(C)-(G) (artery) and 6(H)-(K) (vein) show the corresponding longitudinal sections and vessel diameter analysis of both branches of the bifurcations. The intensity scans of the repetitive measurements are registered and averaged before segmentation. The averaged vessel diameter values at each A-scan location can then be retrieved, with the standard deviation given over several measurements (Fig. 6(D), (F) for the two artery traces and (I), (K) for the two vein traces). The average precision (standard deviation) of the repeated vessel diameter measurements is 3 μm for arteries and 6 μm for veins. Increased variability of diameter measurements is observed in the areas of vessel bifurcations (dashed red rectangles). The higher variability of vein thickness measurements is likely caused by the somewhat poorer visibility of the vein walls which might be related to the somewhat thinner vein walls [35,36].

### 3.3. *In-vivo* measurements: blood flow quantification

**Fig. 7.**
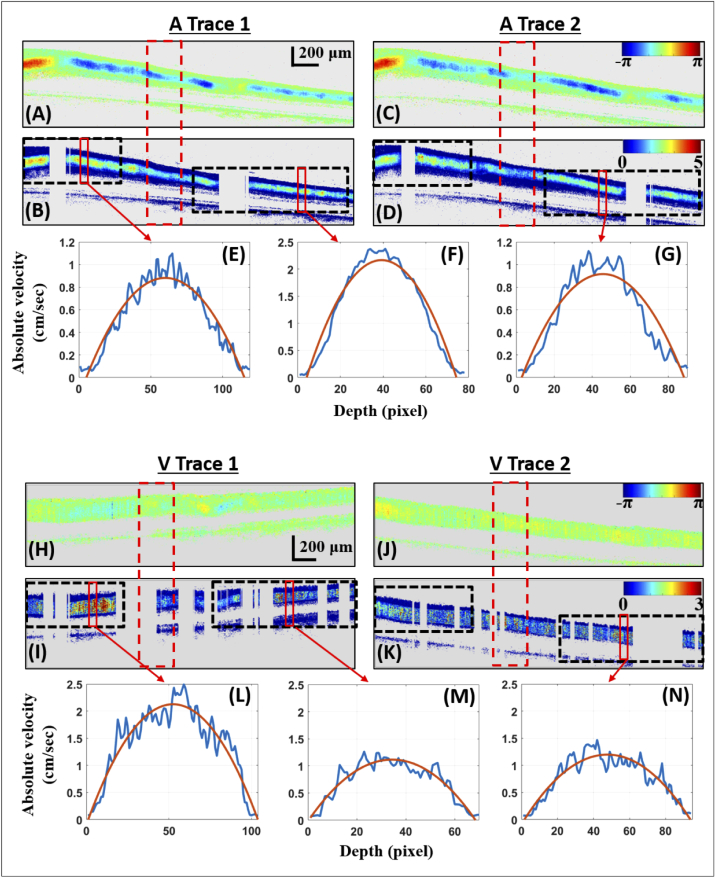
Phase difference tomograms and absolute velocity tomograms of the two traces of the artery and vein bifurcation shown in Fig. 6. For the artery bifurcation, the phase difference tomograms are given in (A) for trace 1 and (C) for trace 2 and their corresponding absolute velocity tomograms in (B) and (D). For the vein bifurcation, the phase difference tomograms are given in (H) for trace 1 and (J) for trace 2 and their corresponding absolute velocity tomograms in (I) and (K). Velocity profile plots at different locations (marked by solid red rectangles on the velocity scans) are given in (E)-(G) for the artery bifurcation and (L)-(N) for the vein bifurcation.

Figure 7 shows bulk motion corrected phase difference tomograms and absolute velocity tomograms of the artery and vein bifurcations of Fig. 6. The total acquisition of the artery bifurcation comprises four cardiac cycles. The scans presented here are taken at the maximum of the systolic phase of the second of these cycles. While scanning along the vessel trace, the curvature of the vessel leads to variation of the Doppler angle. As a consequence, the phase difference values are strongly varying along the vessel as seen in Fig. 7(A), (C). After correction by the Doppler angle, velocity distribution along the vessel appears more homogeneous (Fig. 7(B), (D)). As previously explained, areas at which the Doppler angle is too close to $90^{\circ }$ are not considered and removed from the velocity tomograms (gray areas). The vein Doppler images show only minor pulsations over time; the vein velocity tomograms shown in Fig. 7(I) and (K) are taken at the peak of this small pulse. This effect is further discussed in section 3.4.

For both artery and vein, absolute velocity profiles are presented that correspond to positions before and after the bifurcation (Fig. 7(E)-(G) and (L)-(N)). The profiles are taken as an average over a window of 50 A-scans (indicated by red rectangles). Blood flow is then calculated before and after the bifurcation as an average over the A-scans marked by black dashed rectangles on the velocity tomograms. The values are obtained as an average over 3 cardiac cycles for the artery bifurcation and over the entire acquisition for the vein bifurcation. For the artery bifurcation of Fig. 7, mean inflow is 6.84 ± 0.42 μL/min (from the 3 repetitions of the two traces) and mean outflow is 2.88 ± 0.11 μL/min and 3.88 ± 0.03 μL/min respectively for each of the two branches, or in total 6.76 ± 0.12 μL/min, similar to the inflow, with a percentage difference of 1.2 % (inflow as reference), giving a proof of the consistency of our results. For the vein bifurcation of Fig. 7, mean outflow is 9.10 ± 0.68 μL/min and mean inflow is 3.25 ± 0.6 μL/min and 6.65 ± 0.34 μL/min respectively, for each of the two branches, in total 9.90 ± 0.69 μL/min, similar as the outflow value (within the error margin), with a percentage difference of 8.8 % (outflow as reference). The lower precision of the vein diameter as well as the lower phase difference values, as compared to the artery, may have caused the higher deviation.

Repeating similar imaging of vessel bifurcations in four additional subjects we obtained the results shown in Table 1. The results show good consistency of in- and outflow with a flow difference between 1 and 11 % in 7 of the imaged bifurcations. In two cases (one artery and one vein) the difference was larger, 20 and 27 %. One vein bifurcation could not be evaluated, as the vessel wall could not be sufficiently distinguished from the surrounding tissue. The average diameter (over the repeated measurements) of each vessel is given in Table 2. The arteries imaged range from 65 μm to 116 μm in diameter while the veins imaged range from 77 μm to 142 μm.

**Table 1.** Flow values before and after bifurcation in the imaged vessels of each of the 5 subjects. (Q: blood flow, A: artery, V:vein). For 7 of the imaged vessel bifurcations, the flow percentage difference with respect to the value before bifurcation (given in the last column of the table) is ≤ 15 % (indicated in green). 2 bifurcations have a flow percentage difference ≥ 15 % (indicated in orange). The measurements of one bifurcation could not be evaluated.

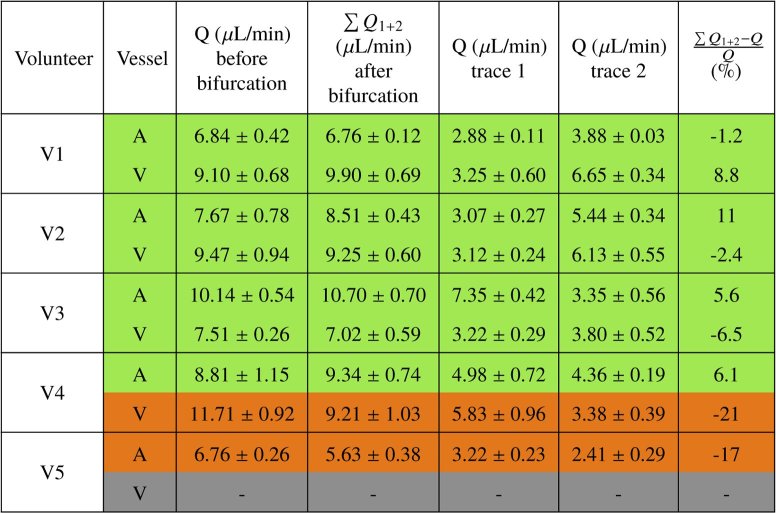

**Table 2.** Diameter values of imaged vessels before and after bifurcation in each of the 5 subjects. (D: diameter, A: artery, V: vein). Color coding is according to Table 1.

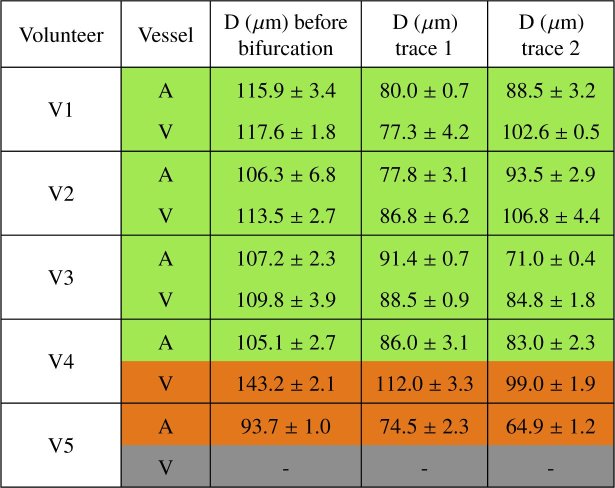

### 3.4. Flow rate over time

**Fig. 8.**
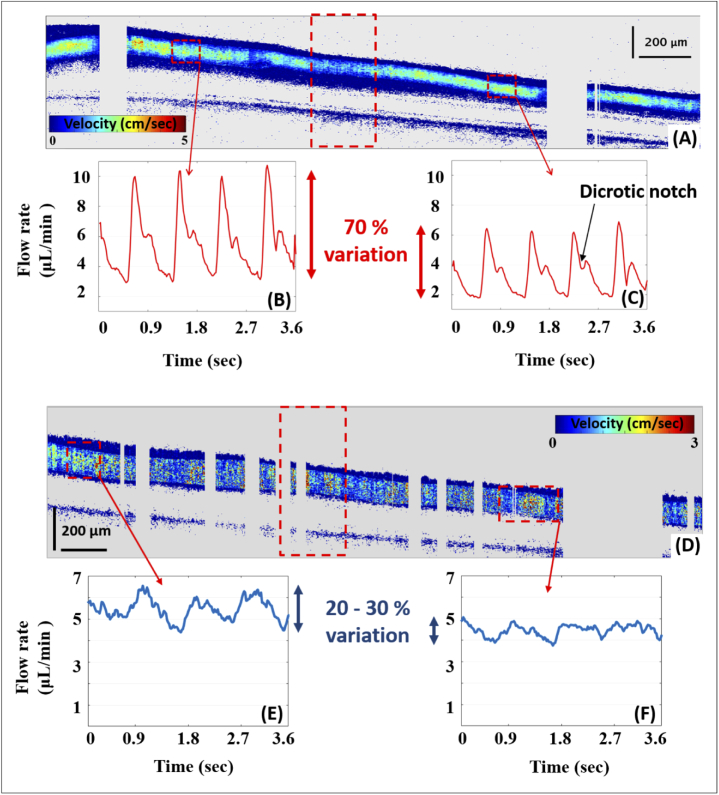
Flow rate of both the artery trace 1 and the vein trace 2 of Fig. 6 measured over time at two distinct locations (marked by small red dashed rectangles) before and after the bifurcation (marked by the large red dashed rectangle). (A), (D) are the absolute velocity tomograms of the two traces. Blood flow pulsation is seen with a variation of about 70 % in the case of the artery (B), (C). A small pulse, with a flow variation up to 30 % is seen in the vein (E), (F). The presented graphs were obtained after averaging over 50 consecutive A-scans. In the case of the artery, pulsation can however also be seen A-scan wise.

Our method offers the advantage to study flow rate over time with a high temporal resolution. As an example, the video of the full acquisition sequence of the first artery trace shown in Fig. 6 is given in supplementary material (Visualization 2). It includes the sequence of the OCT intensity tomograms as well as the corresponding velocity tomograms and is run approximately 3 times slower than the actual acquisition. In the intensity scans, movement inside the vessel can clearly be seen. The dark center line and the vessel wall are also recognizable throughout the entire acquisition. We note that the same speckle are seen in the static tissue (RNFL) over time underlining the good stability of the tracking method. In the velocity images of the artery trace, the different heartbeat cycles are clearly identified. The dicrotic notch (i.e. secondary upstroke in the pulse wave) is also clearly distinguishable. From such imaging, flow rate over time can easily be assessed at different locations. For this same artery trace, variations of the blood flow over time before and after bifurcation are given in Fig. 8(B)-(C). Flow variation is up to 70 % in this case. Flow difference between the dicrotic notch and the maximum of the systolic phase is about 40 %. Similar flow profiles over time are observed on all the imaged retinal arteries. In a similar way, blood flow variations in the veins can be analysed. Figure 8(E)-(F) shows the flow rate over time before and after bifurcation for the second trace of the vein bifurcation shown in Fig. 6. While there are no large pulsations as in arteries, a repetitive pattern is distinguishable. 3 cycles can be seen with a variation of the flow rate up to 20 to 30 %, after averaging over a window of 50 A-scans. The flow variations in veins are too small to be seen on the full sequence of the velocity tomograms.

## 4. Discussion

We introduced a new method to measure absolute blood velocity and flow in retinal vessels that needs only a single OCT sampling beam and retinal tracking. Compared to multi-beam DOCT methods, the method reported in this work has the advantage of less system complexity and simpler alignment. By recording longitudinal sections of the vessels along their trace, employing arbitrary scan patterns, the Doppler angle and vessel diameter can be derived at each position along the vessel trace. A retinal tracker that uses the images of simultaneously acquired LSLO images for motion detection was used to control the OCT sampling beam path to enable repeated measurements along the same vessel trace. This enables the recording of high-quality averaged longitudinal vessel sections. After averaging of multiple B-scans, speckles within static tissue above the vessel (e.g. nerve fiber layer) are not washed out, indicating a lateral repeatability of the B-scans that is better than the extension of a speckle (and thus in the order of the lateral resolution of the system).

**Fig. 9.**
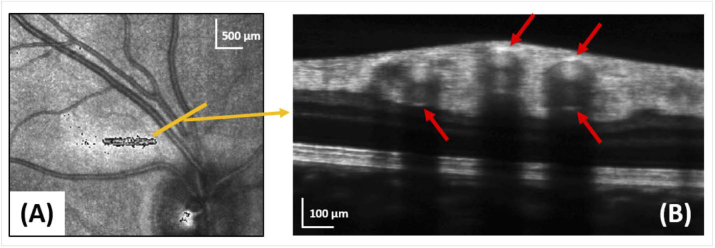
Cross-sectional averaged intensity image (B) of 3 vessels of a healthy subject as shown on the LSLO image (A). The middle artery is the one shown in Fig. 6 before bifurcation.

The method provides both structural and hemodynamic information. The averaged intensity based longitudinal sections provide a very clear identification of the vessel walls, visible as bright lines along the entire course of the vessel trace. This observation is somewhat confirmatory, considering the well-known appearance of vessel cross sections in OCT intensity images (cf. Fig. 9). Sharp bright boundaries are only visible at the very top and bottom of a vessel wall (red arrows in Fig. 9(B)), most of the wall structure along the circumference of the vessels is barely visible at all. This observation indicates that the OCT sampling beam follows the center of the vessel trace with high precision.

Quantitative flow imaging could then first be demonstrated in a simple phantom that allowed comparing the results to the known ground truth. Measured flow values were equal to the set values within 2-3 % for velocities of ∼ 8 - 30 mm/s. For *in-vivo* measurements, a ground truth is not available, however, the results obtained are in agreement with blood flow measured in similar vessels in previous studies with three-beam DOCT systems [20–22]. A direct comparison with laser Doppler velocimetry measurements [37] is in principle possible but beyond the scope of this work. Measurements in vessel bifurcations also provided a check for internal consistency of the results: the in- and outflow to and from the bifurcation was equal within the precision of the results in 7 of 10 cases, with deviations between 1 to 11%, in the range reported for similar measurements by three-beam DOCT systems [20,22,38].

In one case (a vein), the measurement could not be evaluated because the vessel wall could not be reliably differentiated from the surrounding tissue (probably the scan was not carried out exactly along the vessel center). In two cases, the flow mismatch was stronger, 17% and 21%. One reason for this stronger deviation might be related to Doppler angle inaccuracies that could be caused by motion artifacts. It should be noted that artifacts due to axial motion result in a rotation of the B-scan image (axial displacements between entire B-scans are compensated in post processing). These inaccuracies are largely cancelled out by averaging of B-scans that are recorded over a few seconds, as done in our measurements, yielding accurate information on the Doppler angle. However, a related source of error could be image rotations caused by lateral changes of the entrance pupil position that result in a systematic rotation of the image for subsequently recorded B-scans and cannot fully be compensated by averaging. This could, e.g., have occurred between the measurements of the two vessel branches and have led to a different Doppler angle for the two measurements. A systematic error of flow measurements might also have been caused by neglecting the true axial length of the eye, which scales with measured transverse distances. This can slightly distort measured vessel diameters and Doppler angles. In our measurements, a standard eye length has been assumed. This could easily be corrected by precise ocular biometry [39].

Limitations of our method might be associated with the range of vessel diameters that can be measured. If a vessel diameter is too large to be penetrated by the sampling beam, measurements would fail. However, in the healthy eyes that have been investigated we did not encounter any problems associated with insufficient penetration depth into large vessels. Limitations for smaller vessels also have to be considered: up to now, we have only demonstrated measurements in vessels with a diameter > 65 μm. While imaging smaller vessels might be possible, it would pose increasing demands on exact vessel tracing. For example, the determination of the center of the vessel will be more problematic and needs to be improved. Currently, a manual segmentation is performed that can, however, be automatized to further improve the precision of the method. A related problem is residual tracking errors which will have a larger impact on the diameter measurements of a small vessel as the same lateral offset from the center of the vessel (induced for example by a residual tracking error) will lead to a larger error in the measured diameter and consequently to a larger error in the measured flow. As the residual tracking error depends on the fixation capabilities of the subject, a general lower limit of the vessel size for the proposed technique cannot be given. Even though the tracking has a good performance, rapid eye motion as saccades are too fast and cannot be compensated by the tracking. However, these large motions are clearly visible in the recorded B-scans and corresponding images are discarded and excluded from further analysis. Another limitation occurs with vessels that have a Doppler angle close to $90^{\circ }$. We excluded vessel segments with Doppler angle > $85^{\circ }$ from the evaluation because of increasing measurement errors close to perpendicular beam incidence. However, in such a case, beam de-centration at the entrance pupil could help, as this would induce an inclination of the sampling beam at the retinal surface [40].

Another limitation of the present implementation is that we have to manually select several sampling points along the vessel in the LSLO image, which takes ∼ 1 minute. Advanced vessel segmentation methods could improve the situation, e.g., by selecting only the start and end point of the scan along the vessel, the points in between being automatically selected. Finally, the present implementation only provides flow measurements in single vessels, total retinal blood flow measurements are not yet available. This problem could be solved by a software improvement that automatically identifies the major retinal vessels emerging from the ONH that provide the total retinal perfusion. Typically, 9 - 12 vessels carry most of the blood flow. By automatically selecting the points where they emerge from the ONH and choosing a scan pattern that follows their traces along a short distance (e.g. ∼ 1 mm), the flow in these vessels can be measured and added (for arteries and veins separately) to obtain the total RBF.

Applications for our method could comprise diagnosis and follow-up of diseases known to change the RBF [1–4]. In addition to RBF measurements, also the structural images of longitudinal vessel sections could be of interest, e.g., to analyze the vessel diameter in diseases that can change this diameter, such as glaucoma and diabetic retinopathy [41,42]. The method might also enable further investigations of pulsatile diameter changes in spontaneous retinal venous pulsation [43]. Another related application would be the analysis of vessel diameters, as well as plaque depositions at vessel walls, and their association with cardiovascular risk factors [44].

## 5. Conclusion

In this work, we presented a new approach for *in-vivo* measurements of blood flow in retinal vessels that uses a single beam OCT system with an integrated retinal tracker. The method is based on the recording of longitudinal vessel sections along the vessel trace. This approach provides the vessel diameter, Doppler angle, and Doppler flow information simultaneously. Compared to multi-beam systems, the system complexity is reduced and the subject alignment is simplified. Possible applications of the method comprise diagnosis and therapy monitoring of diseases that can alter vessel diameter and blood flow, such as glaucoma or diabetic retinopathy, and cardiovascular diseases. However, the method is not limited to retinal vessel scanning; the arbitrary scan patterns allow imaging along any other retinal structures such as nerve fibers, scars, or other tissue structures.
